# Polygenic risk score prediction of multiple sclerosis in individuals of South Asian ancestry

**DOI:** 10.1093/braincomms/fcad041

**Published:** 2023-02-22

**Authors:** Joshua R Breedon, Charles R Marshall, Gavin Giovannoni, David A van Heel, Genes & Health Research Team, Shaheen Akhtar, Mohammad Anwar, Elena Arciero, Omar Asgar, Samina Ashraf, Gerome Breen, Raymond Chung, Charles J Curtis, Shabana Chaudhary, Maharun Chowdhury, Grainne Colligan, Panos Deloukas, Ceri Durham, Faiza Durrani, Fabiola Eto, Sarah Finer, Ana Angel Garcia, Chris Griffiths, Joanne Harvey, Teng Heng, Qin Qin Huang, Matt Hurles, Karen A Hunt, Shapna Hussain, Kamrul Islam, Benjamin M Jacobs, Ahsan Khan, Amara Khan, Cath Lavery, Sang Hyuck Lee, Robin Lerner, Daniel MacArthur, Daniel Malawsky, Hilary Martin, Dan Mason, Mohammed Bodrul Mazid, John McDermott, Sanam McSweeney, Shefa Miah, Sabrina Munir, Bill Newman, Elizabeth Owor, Asma Qureshi, Samiha Rahman, Nishat Safa, John Solly, Farah Tahmasebi, Richard C Trembath, Karen Tricker, Nasir Uddin, David A van Heel, Caroline Winckley, John Wright, Ruth Dobson, Benjamin M Jacobs

**Affiliations:** Preventive Neurology Unit, Wolfson Institute of Population Health, Queen Mary University of London, London EC1M 6BQ, UK; Preventive Neurology Unit, Wolfson Institute of Population Health, Queen Mary University of London, London EC1M 6BQ, UK; Department of Neurology, Royal London Hospital, London E1 1FR, UK; Preventive Neurology Unit, Wolfson Institute of Population Health, Queen Mary University of London, London EC1M 6BQ, UK; Department of Neurology, Royal London Hospital, London E1 1FR, UK; Blizard Institute, Queen Mary University of London, London E1 2AT, UK; Blizard Institute, Queen Mary University of London, London E1 2AT, UK; Preventive Neurology Unit, Wolfson Institute of Population Health, Queen Mary University of London, London EC1M 6BQ, UK; Department of Neurology, Royal London Hospital, London E1 1FR, UK; Preventive Neurology Unit, Wolfson Institute of Population Health, Queen Mary University of London, London EC1M 6BQ, UK; Department of Neurology, Royal London Hospital, London E1 1FR, UK

**Keywords:** multiple sclerosis, genetics, ethnicity

## Abstract

Polygenic risk scores aggregate an individual’s burden of risk alleles to estimate the overall genetic risk for a specific trait or disease. Polygenic risk scores derived from genome-wide association studies of European populations perform poorly for other ancestral groups. Given the potential for future clinical utility, underperformance of polygenic risk scores in South Asian populations has the potential to reinforce health inequalities. To determine whether European-derived polygenic risk scores underperform at multiple sclerosis prediction in a South Asian-ancestry population compared with a European-ancestry cohort, we used data from two longitudinal genetic cohort studies: Genes & Health (2015–present), a study of ∼50 000 British–Bangladeshi and British–Pakistani individuals, and UK Biobank (2006–present), which is comprised of ∼500 000 predominantly White British individuals. We compared individuals with and without multiple sclerosis in both studies (Genes & Health: *N*_Cases_ = 42, *N*_Control_ = 40 490; UK Biobank: *N*_Cases_ = 2091, *N*_Control_ = 374 866). Polygenic risk scores were calculated using clumping and thresholding with risk allele effect sizes obtained from the largest multiple sclerosis genome-wide association study to date. Scores were calculated with and without the major histocompatibility complex region, the most influential locus in determining multiple sclerosis risk. Polygenic risk score prediction was evaluated using Nagelkerke’s pseudo-*R*^2^ metric adjusted for case ascertainment, age, sex and the first four genetic principal components. We found that, as expected, European-derived polygenic risk scores perform poorly in the Genes & Health cohort, explaining 1.1% (including the major histocompatibility complex) and 1.5% (excluding the major histocompatibility complex) of disease risk. In contrast, multiple sclerosis polygenic risk scores explained 4.8% (including the major histocompatibility complex) and 2.8% (excluding the major histocompatibility complex) of disease risk in European-ancestry UK Biobank participants. These findings suggest that polygenic risk score prediction of multiple sclerosis based on European genome-wide association study results is less accurate in a South Asian population. Genetic studies of ancestrally diverse populations are required to ensure that polygenic risk scores can be useful across ancestries.

## Teaser

Breedon *et al*. report that polygenic risk scores for multiple sclerosis derived from the European-ancestry genetic studies underperform in a South Asian-ancestry population. Their results stress the need for ancestrally diverse genetic studies of multiple sclerosis risk to ensure the generalizability of polygenic risk scores.

## Graphical Abstract

**Graphical abstract:**
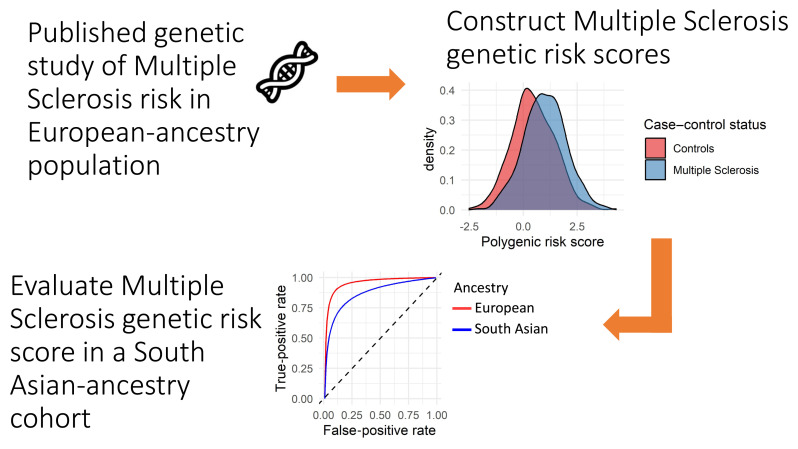

## Introduction

An individual’s risk of developing multiple sclerosis is influenced by common variation across the genome.^1,2^ Multiple sclerosis is a typical complex disease in which the genetic contribution to risk is governed by a large number of susceptibility alleles with individually weak effects. Variation within the major histocompatibility complex (MHC) has the greatest impact on individual risk [odds ratio (OR) associated with DRB1*1501 3.1 and 6.2 for heterozygous and homozygous carriage, respectively].^2,3^ Genome-wide association studies (GWAS) of multiple sclerosis susceptibility have demonstrated at least 200 risk alleles outside the MHC locus, each with a small incremental effect (OR per allele ≤1.3).^2^ There is no convincing evidence for monogenic forms of multiple sclerosis in the general population.^4^

Predicting who is likely to develop multiple sclerosis in the future has potential utility for research studies. Accurate disease prediction could facilitate the design of trials for candidate preventive strategies, such as an Epstein–Barr virus (EBV) vaccine or a vitamin D supplementation trial. As multiple sclerosis is a relatively rare disease, such trials will only have the power to demonstrate a risk reduction if the trial population is sufficiently enriched with people at high risk of multiple sclerosis, effectively increasing the proportion likely to develop the disease.^5^ Furthermore, identifying those at highest risk of disease may allow treatment during the ‘prodromal’ period, prior to overt clinical manifestations.^6^

Polygenic risk scores (PRS) summarize an individual’s cumulative burden of genetic risk alleles to approximate their overall disease risk. Most PRS are calculated by weighting the individual’s burden of risk alleles by the estimated effect of each allele on risk—these estimates are usually obtained from GWAS. In two large cohort studies—UK Biobank (UKB) and University of California San Francisco (UCSF) Expression, Proteomics, Imaging, Clinical (EPIC)—PRS have been empirically demonstrated to distinguish multiple sclerosis cases from controls at a population level.^7-9^

PRS perform poorly in non-European ancestral groups, a phenomenon largely due to differences in linkage disequilibrium (LD) and allele frequencies between populations.^10-12^ It is now clear that multiple sclerosis affects individuals of all ethnic backgrounds and that, broadly speaking, the genetic architecture of multiple sclerosis susceptibility overlaps considerably between ancestral groups.^13-22^ We therefore sought to evaluate the performance of multiple sclerosis PRS in ∼50 000 individuals of South Asian ancestry from the Genes & Health (G&H) cohort to determine the applicability of PRS in this population.

## Methods

### Cohort description and phenotype definition

**Figure 1:**
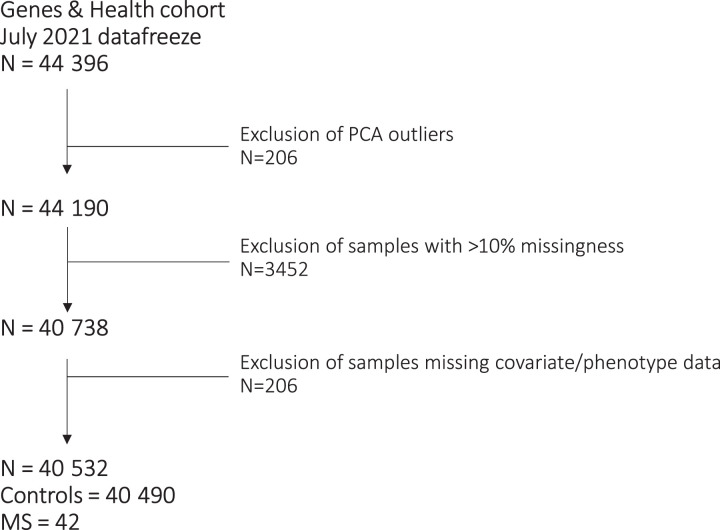
**Flow diagram of individual quality control in G&H.** From an initial 44 396 individuals with genotype data, 40 532 individuals were retained for analysis comprising 42 multiple sclerosis cases and 40 490 controls

We used data from the July 2021 data freeze of G&H, a longitudinal genetic cohort study of ∼50 000 British–Bangladeshi and British–Pakistani individuals.^23^ Genotypes and clinical data for 44 396 participants were included in this study. After exclusion of principal component analysis (PCA) outliers (*n* = 206), samples with >10% missing genotypes (*n* = 3452) and samples without corresponding phenotype/covariate data (*n* = 206), 40 532 individuals were retained for analysis. Of this final cohort, 42 individuals had a coded diagnosis of multiple sclerosis and 40 490 did not (Fig. 1). Cases were defined using linked electronic health records from primary care, hospital episode statistics (HES) data and local hospital recording of admissions and outpatient encounters. Healthcare data were harmonized across International Classification of Diseases Revision 10 (ICD10) codes, Systematized Nomenclature of Medicine (SNOMED) description IDs and SNOMED concept IDs into a consistent three-digit ICD10 coding system. Individuals with at least one multiple sclerosis diagnostic code (ICD10 code G35) in their records were considered cases, and those without were considered non-multiple sclerosis controls. Details of phenotype definitions can be found in Supplementary File 1. An online version of this file is continuously updated and can be viewed here.

### Genotype data and target data quality control

Genomic DNA was obtained from saliva samples using Oragene-600 kits. Individuals were genotyped using the Illumina Global Screening Array chip (version 3) with extra multi-disease content.

Genotypes were imputed using the multi-ancestral Trans-Omics for Precision Medicine Program (TOPMed)-R2 panel. Variant quality control was performed to remove low-quality variants using the following filters: call rate >90%, imputation quality INFO >0.7, minor allele frequency (MAF) >0.01, no deviation from Hardy–Weinberg equilibrium (HWE) at *P* < 1^−10^ and genotype missingness <10%. Sex chromosomes and mitochondrial variants were excluded. Individual quality control was performed to remove PCA outliers and individuals with high missingness. Full details of genotype data quality control can be found in the Supplementary Methods.

### Polygenic risk score calculation

PRS were derived using PRSice-2, which uses a clumping-and-thresholding approach.^9^ We used external weights from the discovery-stage International Multiple Sclerosis Genetics Consortium (IMSGC) 2019 GWAS meta-analysis (cases: 14 802, controls: 26 703).^2^ We used LD-based clumping to determine independent signals using European samples from the 1000 Genomes Project (*n* = 503).^1^ We harmonized single nucleotide polymorphisms (SNPs) between the multiple sclerosis GWAS and the G&H cohort imputed genotype data. LiftOver was used to convert the coordinates to hg38. We excluded SNPs with incompatible alleles and restricted to non-palindromic, biallelic SNPs.

We generated 224 different (but overlapping) PRS by varying the clumping *R*^2^ and *P*-value threshold for variant selection. Many of the SNPs incorporated in these PRS overlap, and so while these PRS are distinct, they are correlated with each other. Specifically, we used clumping R^2^ thresholds of 0.001, 0.01, 0.05, 0.1, 0.2, 0.4 and 0.6 and *P*-value thresholds of 1 × 10^−8^, 5 × 10^−8^, 1 × 10^−7^, 5 × 10^−7^, 1 × 10^−6^, 5 × 10^−6^, 1 × 10^−5^, 5 × 10^−5^, 1 × 10^−4^, 5 × 10^−4^, 1 × 10^−3^, 5 × 10^−3^, 0.01, 0.05, 0.1 and 0.5. For each combination of threshold *P*-value and clumping *R*^2^ value, we derived PRS both including and excluding the MHC region (chromosome 6: 25 000 000–35 000 000 in hg38). In order to isolate the contribution of the MHC, we generated a further 112 scores including just the MHC region. Overall, we generated 336 PRS (112 with MHC, 112 without and 112 just MHC). A null model was generated using the covariates alone [age, sex and genetic principal components (PCs) 1–4]. PRS were calculated using the sum of the weighted allelic burden for each individual, i.e. for the *j*th SNP and the *i*th individual, where G is the genotype dosage and *β* is the effect size of the SNP:


(1) $$\mathrm{PR}S_{i}\;=\;\Sigma G_{ij}\times \beta_{j}$$

Missing genotypes were centred so as to contribute a mean of 0 to the overall score. Discriminative performance was evaluated using Nagelkerke’s pseudo-*R*^2^ metric adjusted for case ascertainment (assuming a population prevalence of 0.002) and corresponding *P*-values, with adjustment for age, sex and the first four genetic principal components. The PRS with the lowest model fit *P*-value was considered the ‘optimal score’, and the statistical significance of this score was evaluated using the *P*-value for the model fit.^24^

Logistic regression was used to determine the OR of multiple sclerosis in each quartile of PRS (compared to the lowest quartile as reference). In all regression models, age, sex and genetic PCs 1–4 were included as covariates. To determine the area under the curve (AUC) discrimination statistics of each PRS and to determine the calibration, we used the fitted probabilities of the logistic models including the PRS as a covariate. We compared the performance of each PRS with null models comprising only age, sex and PCs 1–4.

### UK Biobank replication

To compare the performance of the multiple sclerosis PRS across ancestries, we repeated the analysis using UKB, a longitudinal cohort study of predominantly White British adults aged >40.^25^ We used largely similar methods for SNP and individual quality control. We restricted the analysis to individuals of genetically European ancestry (UKB field ID 22006) determined using principal components. We excluded one of each pair of highly related individuals (kinship coefficient > 0.0884). We generated and tested a variety of PRS using the same methods as in G&H.

To formally compare PRS performance between the two cohorts controlling for sample size bias, we randomly sub-sampled the UKB cohort to have the same number of cases and controls in our G&H analyses (42 cases, 40 490 controls). For each iteration, we determined the optimal PRS and the estimated Nagelkerke’s pseudo-*R*^2^. We calculated an empirical *P*-value for the hypothesis that the liability explained by the PRS was lower in the South Asian-ancestry (SAS) cohort:


(2) $$P\;=\frac{N_{UKB\;<\;GH}+1}{N_{iter}+1}$$

where *N_iter_* is the total number of iterations (1000) and *N*_*UKB* < *GH*_ is the number of iterations in which the observed Nagelkerke’s pseudo-*R*^2^ in the UKB sub-sample was lower than the observed value in G&H. As a comparator, we also evaluated the performance of multiple sclerosis PRS in the whole cohort (without splitting into training and test sets), comprising 2091 multiple sclerosis cases and 374 866 controls. For analyses in UKB, we used the same clumping *R*^2^ values that were optimal in G&H.

### Power calculations

We performed *post hoc* power calculations to determine our statistical power to detect a difference in multiple sclerosis PRS between cases and controls in G&H. To do so, we simulated a normally distributed PRS in 42 cases and 40 490 controls. We varied the difference in the mean of the case and control distributions from 0 to 3 standard deviations. We performed 1000 bootstrap iterations for each scenario and evaluated the power as the proportion of iterations yielding a Wald test *P*-value of <0.05. These simulations showed that, given this number of cases and controls, we would have 91% power to detect a difference of 0.5 standard deviations in the PRS. For context, in UKB participants of European ancestry, the difference in mean PRS between cases and controls is ∼0.7 standard deviations for the MHC PRS and 0.5 standard deviations for the non-MHC PRS.

### Statistical analysis and computing

Analysis of G&H data was conducted within the dedicated Google Cloud Trusted Research Environment. Target data QC was performed using PLINK version >2.^26^ PRS were calculated using PRSice version 2.3.5.^24^ Statistical analysis was performed using R version 4.2.0. Analysis of UKB data was conducted using the Apocrita High Performance Cluster based at Queen Mary University of London.^27^

### Ethical approval

This research was conducted under an approved application to use the G&H resource (‘Brain consortium’ application). G&H was approved by the London South East NRES Committee of the Health Research Authority (14/LO/1240). Replication in UKB was performed under approved application 43101. This research was undertaken under UKB’s existing ethical approval (REC approval 11/NW/0382; North West Multi-centre Research Ethics Committee).

## Results

Following quality control, we analysed data from 40 532 individuals of South Asian ancestry in the G&H cohort, comprising 42 multiple sclerosis cases and 40 490 controls (Table 1). Demographics of included participants are shown in Table 1.

**Table 1:** Genes & Health cohort characteristics

	Control (*N* = 40 490)	Case (*N* = 42)
Sex, *n* (%)		
Female	22 493 (55.6)	30 (71.4)
Male	17 997 (44.4)	12 (28.6)
Age at recruitment, mean (SD)	41.2 (14.2)	41.0 (0.8)
Genetic ancestry, *n* (%)		
British–Bangladeshi	22 900 (56.6)	11 (26.2)
British–Pakistani	17 590 (43.4)	31 (73.8)

**Figure 2:**
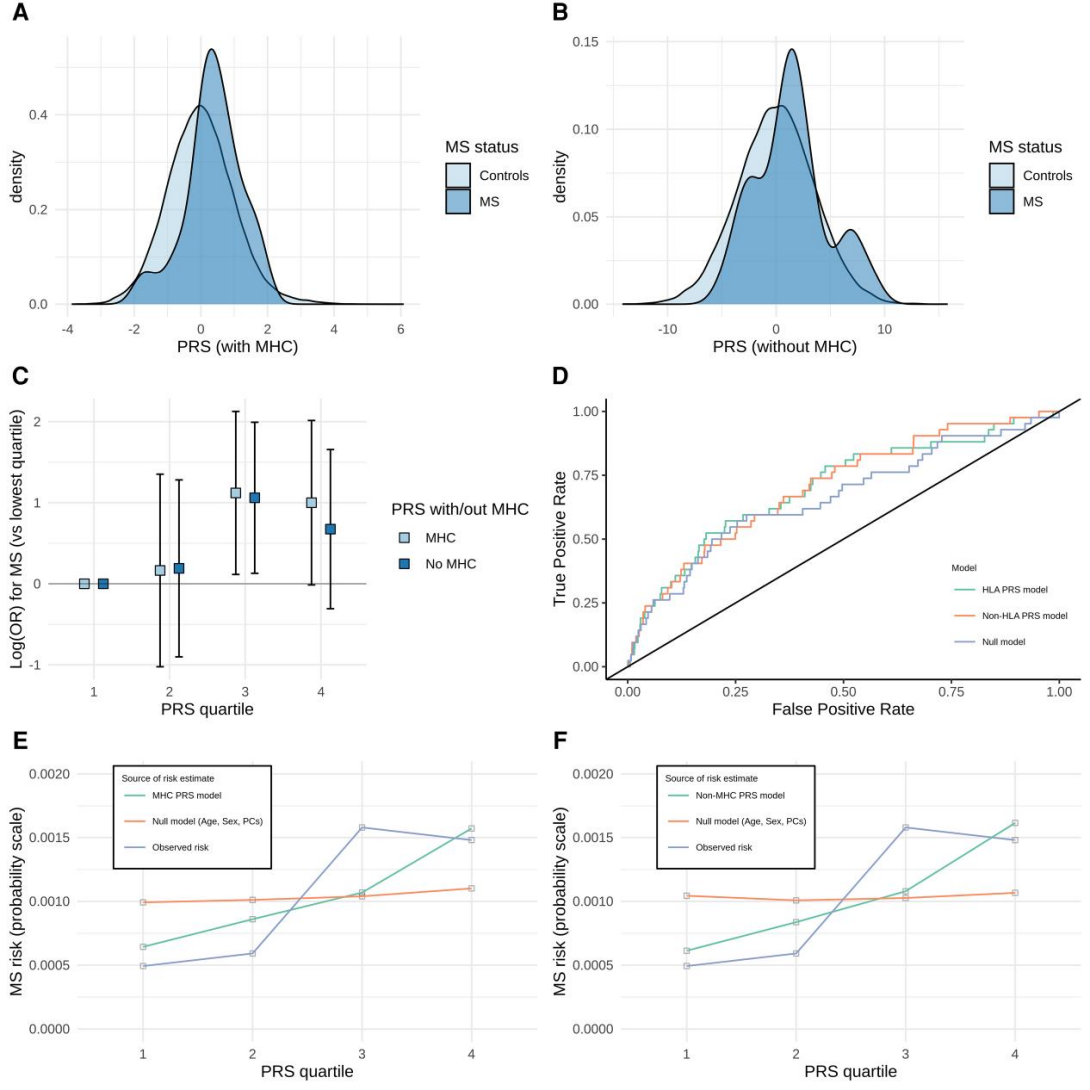
**Multiple sclerosis PRS performance in the G&H cohort of South Asian-ancestry individuals.** (**A** and **B**) Density plots showing the distribution of PRS for PRS with (**A**) and without (**B**) the MHC locus in multiple sclerosis cases and controls. (**C**) Odds ratio quartile plots for individual PRS scores. ORs were calculated relative to the lower quartile. (**D**) Receiver operating characteristic (ROC) curves for the MHC PRS model, non-MHC model and the null model, with corresponding AUC scores. (**E** and **F**) Calibration plots showing the absolute multiple sclerosis disease probabilities (prevalence) for each PRS quartile versus mean fitted probabilities within each quartile from the PRS models. Plots shown for MHC PRS model, non-MHC PRS model, null model and the observed multiple sclerosis risk in each quartile. Odds ratios and AUC values are derived from multivariable logistic regression models

PRS derived from European-ancestry (EUR) multiple sclerosis GWAS were associated with multiple sclerosis in the G&H cohort of British South Asian individuals (*N*_multiple sclerosis_ = 42, *N*_control_ = 40 490) (Fig. 2A and B). The optimal PRS containing the MHC region (PRS_MHC_) explained ∼1.1% of the liability to multiple sclerosis in this cohort (adjusted Nagelkerke’s pseudo-*R*^2^ 0.011, *P* = 0.033, *N*_SNP_ = 1356, clumping *R*^2^ 0.05, threshold *P*-value 0.001). The optimal PRS excluding the MHC region (PRS_Non-MHC_) performed similarly, explaining ∼1.5% of the liability to multiple sclerosis (adjusted Nagelkerke’s pseudo-*R*^2^ 0.015, *P* = 0.015, *N*_SNP_ = 1965, clumping *R*^2^ 0.4, threshold *P*-value 0.001). The difference in performance of the PRS_Non-MHC_ and PRS_MHC_ was not statistically significant (likelihood ratio *P*-value = 1). PRS using variants only lying within the MHC did not correlate with multiple sclerosis disease status (*P* = 0.19).

The predicted risk of multiple sclerosis based on PRS was reasonably well-calibrated to absolute risk (Fig. 2C). Individuals in the top 25% of PRS_MHC_ were nominally more likely to have multiple sclerosis than those in the lowest 25% (OR 2.72, 95% CI 0.99–7.50), although our statistical confidence in this result is tempered by the small number of cases leading to wide confidence intervals which just cross the null. We observed a similar effect for the PRS_Non-MHC_ (OR 1.96, 95% CI 0.74–5.24), again with wide confidence intervals crossing the null. Both the PRS_MHC_ and PRS_Non-MHC_ demonstrated reasonable discrimination between cases and controls at a population level (AUC_MHC_ 0.70, AUC_non-MHC_ 0.71), but it is important to note that age, sex and genetic principal components alone account for much of this discriminative power (AUC_null_ 0.664) (Fig. 2D). In models without any covariates, the PRS_MHC_ and PRS_Non-MHC_ have weaker discriminative ability (AUC_MHC_ 0.63, AUC_non-MHC_ 0.60) but still perform better than chance.

For the PRS_MHC_, in the lowest quartile, 5/10 133 participants had multiple sclerosis (0.05%), as opposed to 15/10 133 in the highest quartile (0.15%). For the PRS_Non-MHC_, 6/10 133 (0.06%) and 12/10 133 (0.12%) had multiple sclerosis in the lowest and highest quartiles, respectively (Fig. 2E and F).

**Figure 3:**
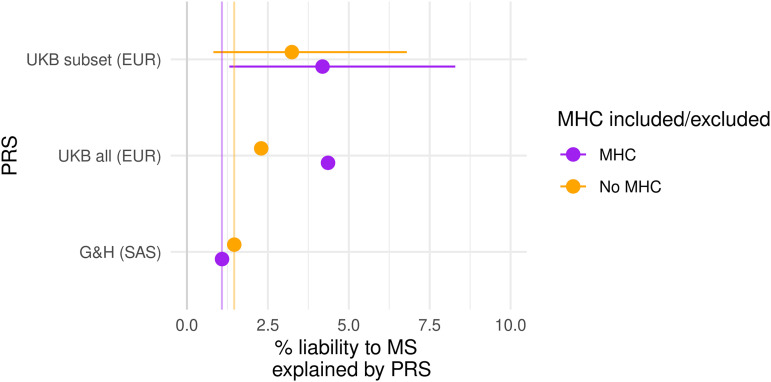
**Estimates of PRS performance in EUR UKB participants and SAS G&H participants.** Each point represents the estimated liability explained by the optimal PRS, with 95% confidence intervals for the sub-samples of UKB. The vertical lines indicate the performance of each score in G&H. PRS containing the MHC are coloured in purple, and those without coloured in orange. Estimates reflect Nagelkerke’s pseudo-*R*^2^ statistic adjusted for disease prevalence, which is derived from multivariable logistic regression models. ‘UKB all’ refers to scores calculated in all EUR-ancestry UKB participants. To control for effects of sample size, we resampled subsets of UKB to have equivalent case and control numbers to G&H (42 cases, 40 490 controls). ‘UKB subset’ refers to estimates derived from 1000 replicates of this random sampling procedure, with empirical 95% confidence intervals

In order to directly compare the performance of the PRS in this cohort with a European-ancestry cohort, we then applied the same methods to UKB (Fig. 3). Using the entire cohort of unrelated EUR-ancestry individuals in UKB (*N*_multiple sclerosis_ = 2091, *N*_Control_ = 374 866), both the PRS_MHC_ and PRS_Non-MHC_ performed better than in G&H, explaining 4.4% and 2.3% of liability, respectively (PRS_MHC_: adjusted Nagelkerke’s pseudo-*R*^2^ = 0.044, *P* = 2 × 10^−211^, *N*_SNP_ = 235454, clumping *R*^2^ 0.4, threshold *P*-value 0.5; PRS_Non-MHC_: adjusted Nagelkerke’s pseudo-*R*^2^ = 0.023, *P* = 1.9 × 10^−104^, *N*_SNP_ = 42759, clumping *R*^2^ 0.6, threshold *P*-value 0.05).

To mitigate the effects of sample size, we randomly sampled 1000 sets of 42 multiple sclerosis cases and 40 490 controls of European ancestry. We used the optimal clumping *R*^2^ thresholds derived from G&H (0.05 and 0.4 for the PRS_MHC_ and PRS_Non-MHC_, respectively). For each iteration, we compared the liability explained in UKB with the observed values in G&H (1.1% for PRS_MHC_ and 1.5% for PRS_Non-MHC_), thus obtaining empirical estimates for the sampling distribution and model fit in UKB.

Using this permutation-based approach, both the PRS_MHC_ and PRS_Non-MHC_ remained strongly associated with multiple sclerosis disease status. In the UKB population, the performance of the PRS_MHC_ was substantially greater than that of the PRS_Non-MHC_, reflecting the large portion of heritability accounted for by this locus. The PRS_MHC_ explained more liability to multiple sclerosis in European-ancestry UKB participants than in G&H (UKB adjusted *R*^2^ 4.3%, 95% CI 1.5–8.5%; G&H adjusted *R*^2^ 1.1%, *P* = 0.01). The difference in the performance of the PRS_Non-MHC_ was less pronounced but also suggestive of weaker performance in the South Asian cohort (UKB adjusted *R*^2^ 3.2%, 95% CI 0.9–6.9%; G&H adjusted *R*^2^ 1.5%, *P* = 0.10), although the confidence intervals span the G&H estimate, and so we cannot reject the possibility that the performance of the PRS_Non-MHC_ is similar in both cohorts.

## Discussion

PRS derived from European GWAS perform poorly in non-European populations across a range of traits and disorders.^10,12^ We report evidence to suggest that this drop-off in PRS performance also applies to multiple sclerosis prediction in a large cohort of South Asian ancestry. We demonstrate that although the European-derived PRS performs relatively poorly in this setting, it does still have some predictive power, consistent with significant overlap in the genetic architecture of multiple sclerosis risk between populations.^13-22^

The lower predictive power of multiple sclerosis PRS we report in an ancestrally South Asian cohort is likely driven by differences in the minor allele frequency of variants and LD structures between European and South Asian populations, rather than due to differences in causal variants.^28^ If variants included in the PRS are not causal themselves but tag causal variants in Europeans, it does not follow that they will tag the causal variant in other populations, diminishing the accuracy of the score. Previous genetic analyses of multiple sclerosis risk in non-European populations—including small studies of South Asian populations—argue that, broadly speaking, the genetic architecture of multiple sclerosis risk between populations is highly correlated.^14,16,17,29^ Our finding that a European multiple sclerosis PRS has some accuracy in a South Asian cohort, but less so than in Europeans, is entirely consistent with this view.

It is notable that the inclusion of the MHC locus did not improve the PRS in the South Asian cohort. This result could be due to limited statistical power, different causal human leukocyte antigen (HLA) alleles and/or poor tagging of causal HLA alleles by the European GWAS variants. It is important to note that available data suggest that the major HLA risk alleles in Europeans have similar effects in South Asians, and so in our view, it is primarily differences in LD (in addition to the limited case numbers) that drive this unexpected result in the cohort, as well as the statistical imprecision of the effect estimates due to the small number of cases in G&H. Larger studies are required to clarify whether this is merely a power issue.

These results should be interpreted with some degree of caution given the relatively small number of multiple sclerosis cases in the G&H cohort (and the resulting wide confidence intervals), the potential inaccuracies of using electronic health records to ascertain cases (including the possibility of missed cases) and the lack of an external validation cohort. Due to the number of multiple sclerosis cases in G&H, we fitted and evaluated the PRS on the same dataset, which increases the risk of overfitting and therefore may produce an inflated estimate of how well the PRS models disease risk in the population. Furthermore, while we aim to compare PRS performance in UKB and G&H, it is important to note that these cohorts were genotyped on different chips and imputed with different panels (TOPMed versus Haplotype Reference Consortium).^25,30^ Therefore, although we use the same external reference panel to perform LD clumping, the SNPs included in the PRS for any given set of clumping-and-thresholding parameters are not identical between cohorts. The mean age in the G&H cohort is also less than that in UKB, raising the possibility of individuals in the G&H control group going on to develop multiple sclerosis in the future. We aimed to mitigate the effect of sample size by sampling the UKB dataset to an equivalent size.

Given the potential uses of a multiple sclerosis PRS in both clinical care and trial design, the limited cross-ancestry transferability of European-derived PRS is concerning and may reinforce pre-existing health inequalities between different ethnic and ancestral groups. Although advances in statistical methods for applying PRS across populations are likely to enhance transferability,^11,31^ there is an unmet need for ancestrally diverse GWAS of multiple sclerosis risk to ensure that genetics can play a useful role in risk stratification.

## Supplementary Material

fcad041_Supplementary_Data

## Data Availability

G&H data are available on application via ​​https://www.genesandhealth.org/research/scientists-using-genes-health-scientific-research. UK Biobank data can be accessed on application via https://www.ukbiobank.ac.uk/. IMSGC. GWAS summary statistics are available on application to the Data Access Committee of IMSGC at https://imsgc.net/. All code used in this study is available on GitHub at https://github.com/benjacobs123456/PRS_UKB_cross_ancestry.

## Appendix: Current Genes & Health Research Team (in alphabetical order by surname)

Shaheen Akhtar, Mohammad Anwar, Elena Arciero, Omar Asgar, Samina Ashraf, Gerome Breen, Raymond Chung, Charles J Curtis, Shabana Chaudhary, Maharun Chowdhury, Grainne Colligan, Panos Deloukas, Ceri Durham, Faiza Durrani, Fabiola Eto, Sarah Finer, Ana Angel Garcia, Chris Griffiths, Joanne Harvey, Teng Heng, Qin Qin Huang, Matt Hurles, Karen A Hunt, Shapna Hussain, Kamrul Islam, Benjamin M Jacobs, Ahsan Khan, Amara Khan, Cath Lavery, Sang Hyuck Lee, Robin Lerner, Daniel MacArthur, Daniel Malawsky, Hilary Martin, Dan Mason, Mohammed Bodrul Mazid, John McDermott, Sanam McSweeney, Shefa Miah, Sabrina Munir, Bill Newman, Elizabeth Owor, Asma Qureshi, Samiha Rahman, Nishat Safa, John Solly, Farah Tahmasebi, Richard C Trembath, Karen Tricker, Nasir Uddin, David A van Heel, Caroline Winckley, John Wright.

## Abbreviations

AUC =: area under the curve
EBV =: Epstein–Barr virus
EPIC =: Expression, Proteomics, Imaging, Clinical
G&H =: Genes & Health
GWAS =: genome-wide association study(ies)
HES =: hospital episode statistics
HLA =: human leukocyte antigen
HWE =: Hardy–Weinberg equilibrium
ICD =: International Classification of Diseases
IMSGC =: International Multiple Sclerosis Genetics Consortium
LD =: linkage disequilibrium
MAF =: minor allele frequency
MHC =: major histocompatibility complex
OR =: odds ratio
PCA =: principal component analysis
PRS =: polygenic risk score(s)
SNOMED =: Systematized Nomenclature of Medicine
SNP =: single nucleotide polymorphism
TOPMed =: Trans-Omics for Precision Medicine Program
UCSF =: University of California San Francisco
UKB =: UK Biobank
