# Supplementary material for: Polygenic risk score prediction of multiple sclerosis in individuals of South Asian ancestry
Source: Brain Commun. 2023 Feb 22;5(2):fcad041. doi: 10.1093/braincomms/fcad041 (PMC10053643; doi:10.1093/braincomms/fcad041)
Supplement: fcad041_Supplementary_Data [file fcad041_Supplementary_Data.zip › Supplementary Material.docx]

**README** for Jul2021_44k_TOPMED-r2_Imputation_b38 dataset

Table of Contents:

GENES & HEALTH

1. GSA chip
2. Genome Studio steps
3. Post genome studio PLINK QC steps 4. Imputation preparation steps
4. TOPMed Imputation
5. Post imputation file merging and filtering
6. Principal Components Analysis for Ethnic Outliers, and KING relatedness analysis 8. Other useful files

9. Running a GWAS or PRS analysis

# GENES & HEALTH

Is a population based study of adult British-Bangladeshi and British-Pakistani individuals (SAS ethnicity). See [www.genesandhealth.org](http://www.genesandhealth.org/) and the Cohort Profile https://doi.org/10.1093/ije/dyz174

DNA was extracted from Oragene saliva samples and genotyped on the Illumina GSA chip.

# GSA chip

The manifest of the Illumina GSAv3EAMD chip (in build 38 format) used by Genes & Health is available here: https://[www.dropbox.com/s/jerphn1qxxuol86/GSAMD-24v3-0-EA_20034606_A2.csv?dl=0](http://www.dropbox.com/s/jerphn1qxxuol86/GSAMD-24v3-0-EA_20034606_A2.csv?dl=0)

There are 730,059 variants/loci on the chip before filtering.

# Genome Studio steps

We performed automated clustering (GenTrain) on n=1970 very high quality call rate samples (selected from an initial 2304 after a preliminary pass).

Additional samples were loaded into the first GenomeStudio Project, of total 8545 non-water samples. Variants were removed based on the following metrics

- low call rate across samples
- variants tagging structural variants
- Chr0, XY and MT variants
- positive HetExcess >0.03
- chitest 100<0.6 (hardy weinberg test)
- AB T Dev, one outlier variant removed
- cluster sep<0.57
- Gentrain score<=0.7

Both automated and manual genotype cloud clustering were reapplied to selected variants, with multiple rounds of increasingly stringent variant and sample filtering. The final dataset had call rate of > 0.992 per female-, and > 0.995 per male-sample across all 637,829 (filtered) variants (which included X and Y chromosomes). This dataset was saved as final ClusterFile4 12Dec2019.egt file and then applied to subsequeny SubProjects. There are ~12,000 variants which are duplicates at the same position, with the same alleles (sometimes of different assay designs). These have slightly different variant names. These have not been removed as they are useful for some QC (e.g. intra-assay reproducibility). We suggest these duplicate variants are removed for most analyses (they are only in the chip data, they are not in the imputed data). There are lots of variants that are non-polymorphic, these were kept in as some may be polymorphic in subsequent samples.

This ClusterFile4 was then applied to subsequent GenomeStudio Projects of further samples. GenomeStudio can only cope with ~8000 samples (*600k variants) due to memory issues even on the highest spec PC. Therefore multiple Projects were created, and the output plink files from each Project merged into one dataset (12 Projects for the 44k dataset, from July 2019 to June 2021). We did not observe evidence for major batch effects/cluster drift over this period:

| Subproj1 | total genotyping rate 0.999446 | 637829 variants * 8023 people (of 8545 non-water samples attempted) |
| --- | --- | --- |
| Subproj2 | total genotyping rate 0.999438 | 637829 variants * 6427 people (of 7029 non-water samples attempted) |
| Subproj3 | total genotyping rate 0.999426 | 637829 variants * 4830 people (of 5130 non-water samples attempted) |
| Subproj4 | total genotyping rate 0.999396 | 637829 variants * 2939 people (of 3134 non-water samples attempted) |
| Subproj5 | total genotyping rate 0.999401 | 637829 variants * 2927 people (of 3135 non-water samples attempted) |

--------Feb 2020 Data Freeze--------

Subproj7 total genotyping rate 0.999337 637829 variants * 4726 people (of 5127 non-water samples attempted) Subproj8 total genotyping rate 0.999366 637829 variants * 2538 people (of 2729 non-water samples attempted)

---------Nov 2020 Data Freeze-------

| Subproj9 | total genotyping rate 0.999421 | 637829 variants * 4665 people (of 4965 non-water samples attempted) |
| --- | --- | --- |
| Subproj10 | total genotyping rate 0.999379 | 637829 variants * 1792 people (of 1899 non-water samples attempted) |
| Subproj11 | total genotyping rate 0.99961 | 637829 variants * 1984 people (of 2090 non-water samples attempted) |
| Subproj12 | total genotyping rate 0.999496 | 637829 variants * 1961 people (of 2100 non-water samples attempted) |

---------Jul 2021 Data Freeze-------

Further description in the methods of this paper: https://doi.org/10.1016/j.cell.2020.06.045

# Post genome studio PLINK QC steps

Various metrics (call rate, gender fails, etc) by chip, saliva aliquot plate, UK Biocentre plate, were inspected to identify clusters of bad samples due to lab error. One chip with multiple failing samples (203884520041) was entirely removed.

Intersex samples: there are 10 samples with suspected Klinefelters. 4 of these are known Klinefelters from NHS health records. 1 individual has taken part twice. These samples have not been removed from the data.

15001903138117 205166820018_R07C01

15001801321616 205090820048_R07C01

| 15001801335276 | 205084670025_R04C01 |
| --- | --- |
| 15001601140319 | 203764440133_R05C01 |
| 15001502033766 | 203807910056_R08C01 |
| 15001601141726 | 203760310107_R03C02 |
| 15001801323503 | 203765340096_R04C02 |
| 15001801323206 | 203765340108_R01C01 |
| 15001506220376 | 203807900114_R04C02 |
| 15001506225143 | 203764430031_R07C01 |

Various other sample level quality checks were performed, and samples excluded where the error could not be simply resolved. We were conservative and removed samples if there was any doubt.

- stated gender versus DNA gender fails
- exome sequence versus GSA data fails
- duplicate GSA genotypes that should not be sample duplicates
- samples that should be duplicate but are not in GSA genotypes
- a few late consent withdrawals

Only chip genotyped samples with valid NHS numbers were then kept.

Where there were duplicate (triplicate etc) chip genotyped samples with the same NHS number, samples with the highest call rate were kept. These could be duplicate either because of the same NHS number with different Oragene barcodes (i.e. the volunteer has taken part in the study twice) or because of deliberate repeated chip genotyping.

However 7 pairs of individuals were kept who looked like identical twins. Each had different NHS numbers. A decision was taken not to recontact these individuals, and therefore identical twin status (as opposed to multiple NHS numbers for an individual - which was not suggested by health records) could not be absolutely confirmed. These individuals are kept in the dataset.

| 15001502030134 | 15001502030648 |
| --- | --- |
| 15001605211501 | 15001601143108 |
| 15001608194908 | 15001612100257 |
| 15001612102057 | 15001612102090 |
| 15001801322277 | 15001801322297 |
| 15001801332058 | 15001903130633 |
| 15001608191359 | 15001608191352 |

# Imputation preparation steps

Input file was plink --

bfile=**2021_07 GNH GSAv3EAMD SubProj1to12 ClusterFile4 PostQC ValidNHSonly with EthnicOutliers**

Removed monomorphic SNPs AND nonACGT variants

plink2 --snps-only just-acgt --maf 0.0000000000000000000000000000000001 Removed palindromic SNPs (A/T, T/A, C/G, G/C) and removed chrY

Uploaded the file to TOPMED QC check stage to get SNPs with strands to flip flipped the strands in plink

For X chr variants with >30 het haploid genotype calls, removed the variant completely Set all other het haploid X chr genotypes to missing.

Finally, variants with MAF<0.0001 were excluded.

The prepared plink files were uploaded and run on TOPMED-r2 Minimac4 1.5.7, in 2 batches Output file plink --

bfile=**2021_07 GNH GSAv3EAMD SubProj1to12 ClusterFile4 PostQC ValidNHSonly with EthnicOutliers_snpsACGT_NonMono_NonPalin_maf0.0001_strandflips_nohh_NotChrY**

# TOPMed Imputation

The prepared plink files were uploaded and run on the TOPMed-r2 Minimac4 1.5.7 Imputation Server, in 2 batches (https://imputation.biodatacatalyst.nhlbi.nih.gov/#!).

Samples in the batches were selected at random using random number generator i.e. the mix of British- Bangladeshi, British-Pakistani should be similar in each batch.

Rsq filter of 0.3 was applied to each batch within the Imputation Server.


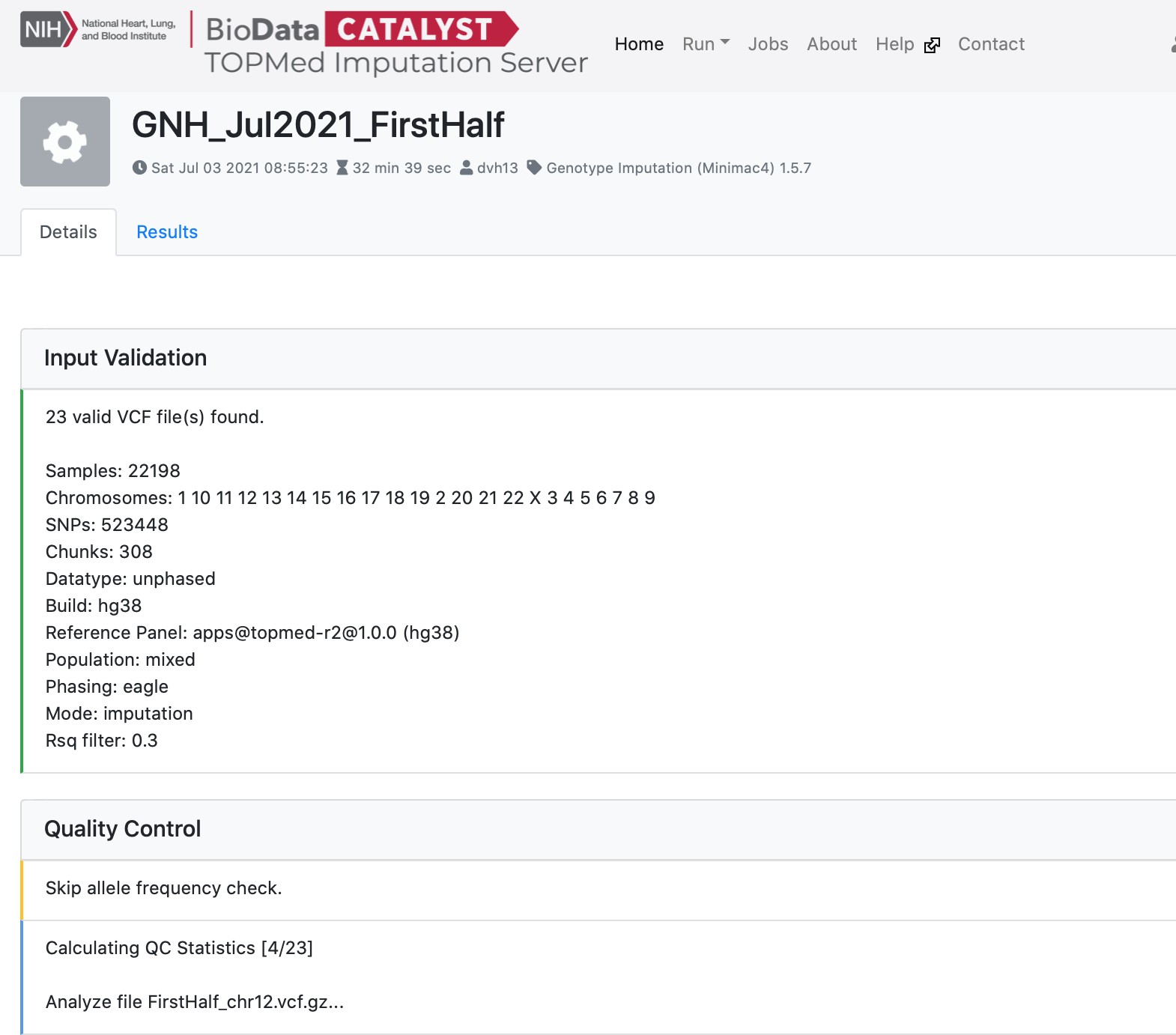


Because the very large new TOPMED panel is reported to impute MAF=0.001% variants accurately (whether this is true for SAS ethnicity is not well tested) we did not apply a MAF filter to the output.

Output saved in folder name: **vcf_topmed-r2_rawbatches_unMAFfiltered**

Relabelled chrX as chr23

Relabelled folders: FirstHalf as Batch01, LastHalf as Batch02

# Post imputation file merging and filtering

Run on QMUL Apocrita HPC:

(across all individual chr 1..23)

Update notes for **version03** of merged vcfs/bgens:

- Compared to version 1, the bcftools --missing-to-ref command was deleted: files will contain missing genotypes
- There was confusion in version 1 with some files being labelled as MAF>0.0001 and others MAF>0.00001
- In version 2 MAF>0.00001 filtering is used
- Due to a bug in bcftools, in version1 some variants present in Batch01 but not in Batch02 were set as all reference genotypes in Batch02.
- In version 3, complex multiallelic variants at a site are collapsed into (mutliple) simple biallelic variants and only those present/imputed in both Batch01 and Batch02 imputation are included.
- No need to compute the intersection between the batches (bcftools isec), which is very slow and doesnt quite do what we want with multiallelic variants.

The 2 batches of TOPMED output were merged, and info/statistics recomputed: ( using bcftools/1.13 )

bcftools merge ../vcf_topmed-r2_rawbatches_unMAFfiltered/Batch01/chr"$ii".dose.vcf.gz ../vcf_topmed-

r2_rawbatches_unMAFfiltered/Batch02/chr"$ii".dose.vcf.gz --merge none --threads 8 -Ou | bcftools plugin impute-info --threads 8 -Ou | bcftools plugin fill-tags -Oz

--threads 8 -- -t AC,AN,AF,MAF,F_MISSING >chr"$ii".dose.merged.vcf.gz

The merged vcfs were filtered, then indexed, then variant info summary files made:

bcftools view chr"$ii".dose.merged.vcf.gz -i 'MAF > 0.00001 && INFO > 0.3 && F_MISSING <0.2' --threads 8 -Oz -o chr"$ii".dose.merged_INFO0.3_MAF0.00001_F_MISSING0.2.vcf.gz

bcftools index --csi chr"$ii".dose.merged_INFO0.3_MAF0.00001_F_MISSING0.2.vcf.gz

bcftools query -f '%ID\t%CHROM\t%POS\t%REF\t%ALT\t%AF\t%MAF\t%AC\t%F_MISSING\t%INFO/INFO\n' chr"$ii".dose.merged_INFO0.3_MAF0.00001_F_MISSING0.2.vcf.gz > chr"$ii"_variant_info.tab

Further files were made from the vcfs: bgen and bgen-index

qctool_v2.0.8-CentOS_Linux7.6.1810-x86_64/qctool \

-g chr"$ii".dose.merged_INFO0.3_MAF0.00001_F_MISSING0.2.vcf.gz \

-og chr"$ii".dose.merged_INFO0.3_MAF0.00001_F_MISSING0.2.8bit.bgen \

-vcf-genotype-field GP -ofiletype bgen_v1.2 -bgen-bits 8 -threads 8

( using bgen/1.1.6 )

bgenix -g chr"$ii".dose.merged_INFO0.3_MAF0.00001_F_MISSING0.2.8bit.bgen -index

Outputs saved in folder name:

QMUL apocrita HPC: /data/Blizard-VanHeelLab/GenesandHealth/GSAv3EAMD/Jul2021_44k_TOPMED- r2_Imputation_b38/topmed/topmed-r2_merged_version03/

**Google Cloud Trusted Research Environment:**

**gs://qmul-sandbox-production-library-red/genesandhealth/GSAv3EAMD/Jul2021_44k_TOPMED- r2_Imputation_b38/topmed-r2_merged_version03/**

# Principal Components Analysis for Ethnic Outliers, and KING relatedness analysis

(analyses done by Teng Heng, Wellcome Sanger Institute)

- The ELGH cohort consists of 44396 individuals
- 355862 variants remained after the following filtering steps:
  - include only autosomal (chr 1-22)
  - include only common variants (MAF > 0.01)
  - include only call rate >99%
  - include only variants that passed HWE in declared Bangladeshi individuals
- KING was run to estimate pairwise relationships up to 4 degrees.
  - Final output: "**GH_44k_autosome_maf0.01_geno0.01_hwe1e-6_relatedness_estimation.kin0**"
- From relationship pairs 3rd and closer:
  - We identified a minimal set of 14728 individuals who had at least one relative that may need to be removed from some analyses, leaving 29668 individuals. This was done in such a way as to minimise the number of individuals being removed i.e. starting by removing the person with the most relatives, then the next most etc. Analysts may wish to recreate these lists taking into consideration which individuals have the phenotype data they need, or are cases for a particular disease.
- Returning to the variants, 353140 remained after excluding palindromic SNPs
- Strand flipping performed on relevant variants in the ELGH cohort
  - 175237 flipped
- ELGH variant IDs standardised to match with reference cohort
- The reference cohort consists of 3433 individuals from 1000G and HGDP
  - 349632 filtered ELGH variants can be found in the reference cohort
- Further exclude 1285 variants (348347 remaining)
  - Due to missingness and AF differences in ELGH and reference South Asians
- LD pruning and excluded long LD ranges (243795 of 348347 variants removed)
  - Window size 1000kb
  - Step size 50
  - LD r2 cutoff 0.1
  - long LD regions (https://github.com/meyer-lab-cshl/plinkQC/blob/master/inst/extdata/high-LD-regions- hg38-GRCh38.txt)
- PCA up to 50 PCs performed on the 3433 reference individuals, 104552 variants.
  - 44396 ELGH individuals projected into PCA
- UMAP with 7PCs visually seperates the reference super populations neatly
  - Highest combined sensitivity and specificity for inferring Bangladeshi and Pakistani ethnicities compared to UMAPs with 5-9PCs
- Using UMAP with 7PCs, inferred Bangladeshi and Pakistani individuals, and excluded 76 outliers ("3others").
- In the remaining 44320 ELGH individuals, repeated PCA on unrelated individuals, projecting related individuals into the PC, 104552 variants. (29619 unrelated and 14701 related after excluding 76 outliers)
  - The PCs have been included in columns [8-57] of "GH.44k.declared_and_inferred_ethnicities.50PCs.txt"
- UMAP with 4 PCs identifies distinct Pakistani and Bangladeshi clusters without teasing out excessive population structure.
  - 190 individuals with discrepant inferred ethnicities, including 130 outlier individuals that do not fall into the main Bangladeshi and Pakistani clusters. 60 individuals have switched inferred ethnicities from the earlier PCA/UMAP using reference datasets, and they do not form a cluster on the UMAP from the PCA using reference dataset.

**PCA1.pdf The first PCA done on reference populations (1000G and HGDP), with Genes & Health individuals projected into it. UMAP with 7PCs plotted to exclude non-SAS_outliers from further analysis.**


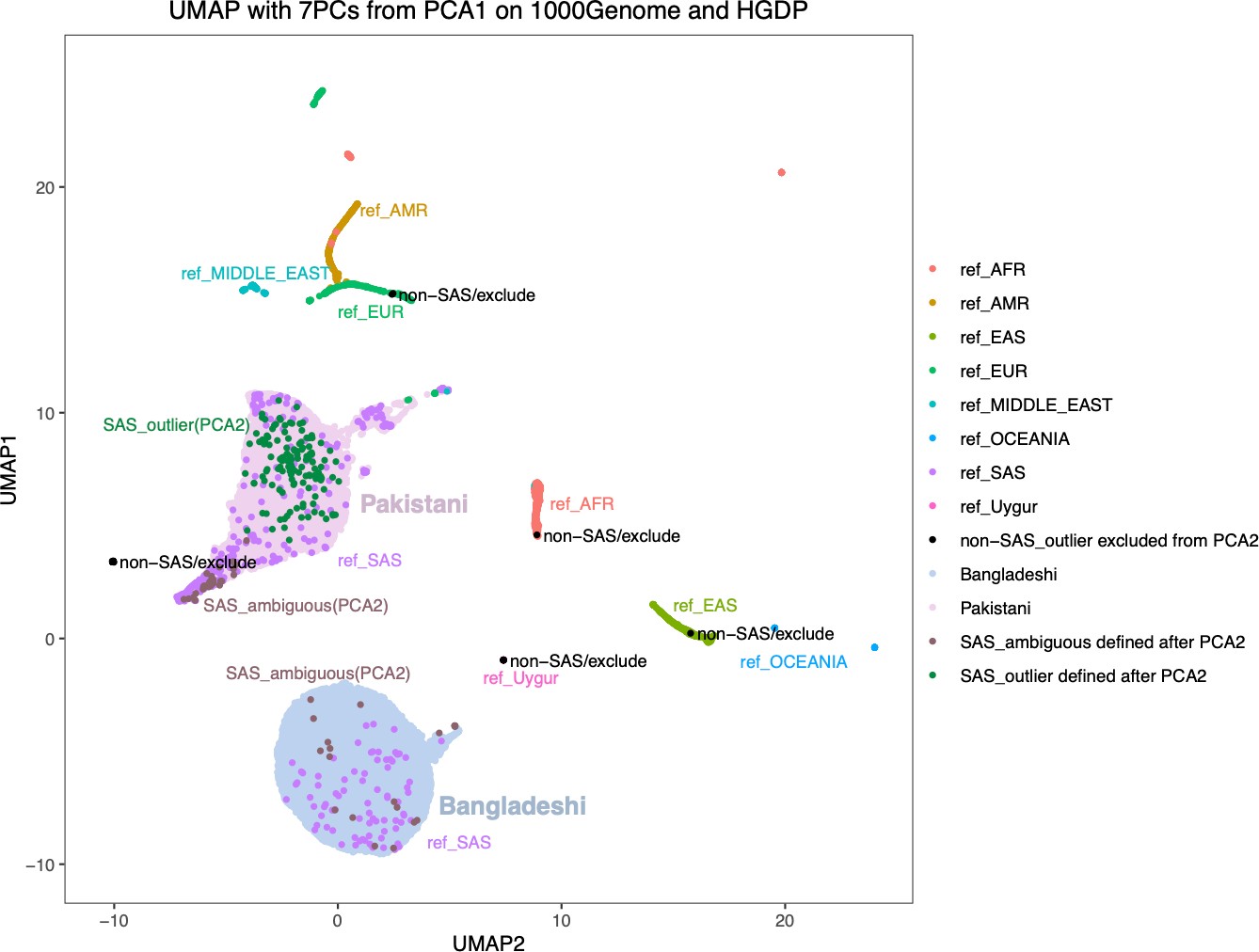


**PCA2.pdf The second PCA done on Genes & Health individuals (after excluding non- SAS_outliers). UMAP with 4PC was plotted to determine SAS_outliers.**


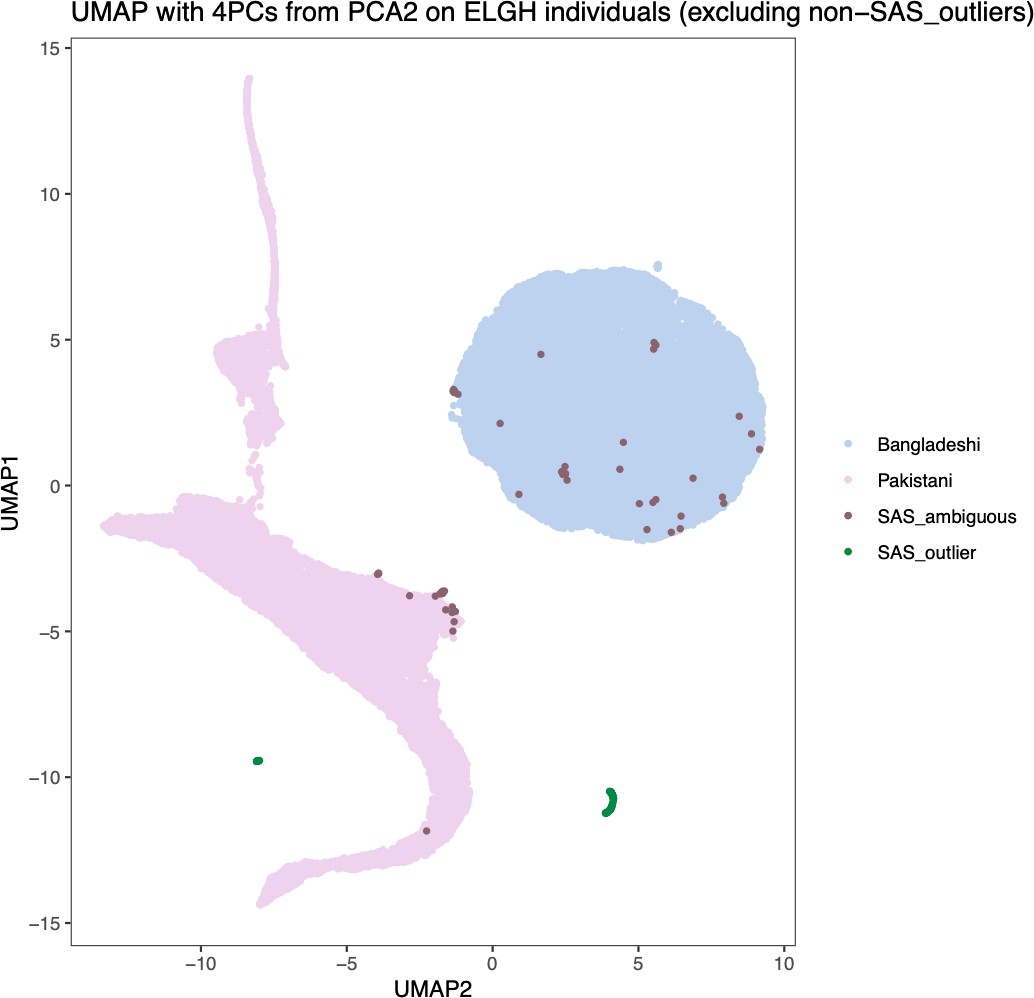


Suggested to remove 130 SAS_outlier samples + 76 non_SAS_outlier samples, so:

Final ‘Jul2021 44k’ dataset contains:

**n=44190 without ethnic outliers** (ie for use in GWAS) (26537 British-Bangladeshi, 17653 Pakistani - as defined by PCA2)

(see below section 9 for premade covariate file with these PCs: GNH.44190.noEthnicOutliers.covariates.20PCs.withS1QST_diabetes.txt )

**n=44396 with ethnic outliers** (ie for use in PRS and other analyses, Recall studies, etc)

# Other useful files

A LD=pruned genetic relatedness matrix (GRM) for use in SAIGE was made from the direct-GSAchip- genotyped dataset (see step 4) as follows:

PLINK v2.00a3LM

--bfile

2021_07 GNH GSAv3EAMD SubProj1to12 ClusterFile4 PostQC ValidNHSonly withEthnicOutl iers_snpsACGT_NonMono_NonPalin_maf0.0001_strandflips_nohh

--geno 0.01

--indep-pairwise 500 50 0.2

--maf 0.01

These variants were pruned out, and the plink GRM filenames are:

--bfile bfile_forSAIGEgrm_44396_chipgenotypes_indep-pairwise_500_50_0.2_LDpruned_NotChrY

# Running a GWAS or PRS analysis

The vcf and bgen files are relatively lightly filtered. Most users will want to apply more stringent filters to call rate, INFO, and MAF before interpreting GWAS or other results. Beware if you do not!

We have premade a covariate file GNH.44190.noEthnicOutliers.covariates.20PCs.withS1QST_diabetes.txt

With fields:

OrageneID SAMPLEID S1QST_Diabetes S1QST_Gender S1QST_YearBirth LABCO_YearInLab AgeAtRecruitment AgeAtRecruitment_Sq inferred_elghPCA

| inferred_elghPCA_ethnicity PC1 PC2 | PC3 PC4 PCPC6 | PC7 PC8 | PC9 |
| --- | --- | --- | --- |
| PC10 PC11 PC12 PC13 PC14 PC15 | PC16 PC17 PC18 PC19 | PC20 |  |

**S1QST_Diabetes** is self-stated diabetes no (0) or yes (1) from the stage 1 questionnaire. This is mostly type 2 diabetes with a bit of type 1 and other. However it should give a HUGE signal at TCF7L2 which is the major type 2 diabetes locus. Please use this as a positive control in your GWAS analyses. If you don’t see something like a P=10-30 signal around chr10:112998590:C:T then you have done something wrong!

**S1QST_Gender** is self-stated gender with corrections for DNA gender and any questionnaire mis-data- entry cleaned up.

**S1QST_YearBirth** is year of birth from the questionnaire. **LABCO_YearInLab** is year the sample arrived in the lab **AgeAtRecruitment** is LABCO_YearInLab - S1QST_YearBirth

**inferred_elghPCA_ethnicity** is 1 for Bangladeshi and 2 for Pakistani by tight PC ethnicity.

A file will also be provided to connect pseudoNHS numbers (for health record phenotypes) with OrageneID

/ GSA chipID (for DNA sample based genotypes).

David van Heel Karen Hunt Teng Heng Qinqin Huang

Genes & Health Research Team

-- last update date as filename --
